# Risk factors for chronic kidney disease and septic shock with hypertension in adults and children

**DOI:** 10.3389/fneph.2025.1671763

**Published:** 2025-10-22

**Authors:** R. Mohamad Javier, Jonathan Salim, Bethari Lekso Aji, Benardinus Prima Ardjie Pradipta, Choirin Nur, Iqbal Muhammad, Livaldi Naufal Aflah, Immaculata Agata Bornok Rettauli, Cristal Audrey, Irma Wijayaningtyas, Yosua Darmadi Kosen, Adhitya Fajriyadi, Nourma Sabila, Fernando Pangruruk Salipadang, Mahardika Adhitya Nugraha, Nadhira Yuisi Cheda, Andra Purwanto Yogatama Putra, Dhial Falah Mahasin, Mutiara Delia Subiyanto, Arkan Zikri Berlian, Muhamad Zulfikar Hadiaturahman, Ratna Kumala Luthfi, Muhammad Reva Aditya, Hafidha Camila Arif, Kristian Kurniawan

**Affiliations:** ^1^ Department of Cardiology and Vascular Medicine, Universitas Indonesia Hospital, Depok, Java, Indonesia; ^2^ Kalideres Regional General Hospital (RSUD Kalideres), Jakarta, Indonesia; ^3^ Dharmais National Cancer Center Hospital, Jakarta, Indonesia; ^4^ Lavalette Hospital, Malang, Indonesia; ^5^ Haji Batu Mother & Child Hospital, Batu City, Indonesia; ^6^ Faculty of MedicineMuhammadiyah University of Malang, Malang, Java, Indonesia; ^7^ Nurussyifa General Hospital, Kudus, Indonesia; ^8^ Duta Mulya General Hospital, Bekasi, Java, Indonesia; ^9^ Altius Hospitals Harapan Indah, Bekasi, Java, Indonesia; ^10^ EKA Grand Family Mother and Child Hospital (RSIA EKA Grand Family), Tangerang, Banten, Indonesia; ^11^ Prembun Regional Hospital (RSUD Prembun), Kebumen, Java, Indonesia; ^12^ Faculty of Medicine, Jenderal Soedirman University, Purwokerto, Java, Indonesia; ^13^ Faculty of Medicine, Maranatha Christian University, Bandung, Java, Indonesia; ^14^ Faculty of Medicine, Tanjungpura University, Pontianak, Kalimantan, Indonesia; ^15^ Siloam Hospital, Jember, Indonesia; ^16^ Faculty of Medicine, Sam Ratulangi University, Manado, Sulawesi, Indonesia; ^17^ Division of Psychosomatic and Palliative Medicine, Department of Internal Medicine, Cipto Mangunkusumo National General Hospital (RSCM), Jakarta, Indonesia; ^18^ Faculty of MedicineSultan Agung Islamic University, Semarang, Java, Indonesia; ^19^ Faculty of Medicine, Indonesian Christian University, Jakarta, Indonesia; ^20^ Faculty of Medicine, Gadjah Mada University, Yogyakarta, Indonesia; ^21^ Dr. Sardjito Hospital, Yogyakarta, Indonesia; ^22^ Faculty of Medicine, UPN Veteran Jakarta, Jakarta, Indonesia; ^23^ Faculty of Medicine, Sultan Agung Islamic University, Semarang, Java, Indonesia; ^24^ Division of Endocrinology, Metabolism and Diabetes, Department of Internal Medicine, Cipto Mangunkusumo National General Hospital (RSCM), Jakarta, Indonesia; ^25^ Aisyiyah Klaten General Hospital (RSU Aisyiyah Klaten), Klaten, Java, Indonesia; ^26^ Faculty of Medicine, Brawijaya University, Malang, Java, Indonesia; ^27^ Faculty of Medicine, Maulana Malik Ibrahim State Islamic University, Malang, Indonesia; ^28^ Faculty of Medicine and Science, Universitas Katolik Indonesia Atma Jaya, Jakarta, Indonesia

**Keywords:** chronic kidney disease (CKD), hypertension, sepsis, septic shock, fluid management, central venous pressure (CVP)

## Abstract

**Background:**

Chronic kidney disease (CKD) affects nearly 10% of the global population and often progresses silently to end-stage renal disease, requiring dialysis or transplantation. Hypertension, prevalent in both adults and children, is a key driver of CKD progression. Acute kidney injury (AKI), particularly sepsis-associated AKI (S-AKI), poses a critical risk for long-term renal dysfunction, especially in patients with pre-existing CKD. S-AKI, defined by abrupt renal function decline during sepsis or septic shock, can accelerate CKD progression, yet its risk factors and outcomes across pediatric and adult populations remain incompletely characterized.

**Objective:**

Aims to systematically evaluate existing research on the relationship between Risk Factors for CKD and Septic Shock with Hypertension in Adults and Children.

**Methods:**

A systematic literature search was conducted using PubMed, Google Scholar, and the Cochrane Library for studies published between 2004 and 2024. Search terms included “chronic kidney disease,” “septic shock,” “hypertension,” and “acute kidney injury.” After applying PRISMA-based screening and eligibility criteria, 9 studies were included for qualitative synthesis.

**Results:**

A total of 762 articles were identified through database searching. After screening and eligibility assessment, 9 studies were included in the final synthesis. The findings revealed that both CKD and hypertension are significant independent risk factors for S-AKI and septic shock. Preexisting albuminuria, uncontrolled blood pressure, advanced age, and diabetes mellitus were frequently associated with poor outcomes. Several studies highlighted the role of MPP and fluid resuscitation strategies in preventing AKI progression in septic patients. In pediatric populations, a history of AKI was strongly associated with new-onset hypertension and subsequent CKD development, increasing vulnerability to severe septic complications.

**Conclusion:**

CKD and hypertension significantly increase the risk of septic complications and worsen renal outcomes, particularly in patients with fluid management challenges. Early identification of high-risk patients, individualized hemodynamic targets, and tailored fluid resuscitation strategies are critical in reducing morbidity and mortality. Special attention is needed in pediatric patients with limited nephron reserve, where long-term surveillance and early intervention may improve outcomes.

**Systematic Review Registration:**

https://www.crd.york.ac.uk/prospero/display_record.php, identifier PROSPERO (CRD420251146866).

## Introduction

Chronic kidney disease (CKD) affects 9.1% of the global population and often progresses silently to end-stage renal disease, requiring costly interventions like dialysis or transplantation (1). CKD is defined by structural or functional renal abnormalities lasting over three months (2). Hypertension significantly contributes to CKD progression; hypertensive men have a 2.14-fold and women a 1.54-fold increased risk of developing CKD (3). In children, hypertension is rising, affecting approximately 4% of those under 19 years, with a steadily increasing prevalence over recent decades. Unlike in adults, pediatric hypertension is defined using blood pressure percentiles adjusted for age, sex, and height, making it essential to consider these variables when establishing management thresholds and hemodynamic targets such as mean arterial pressure (MAP).

Acute kidney injury (AKI), common in critically ill and hospitalized children, is a notable risk factor for pediatric hypertension and subsequent CKD. High-risk groups include those with sepsis, cardiac surgery, malignancies, or nephrotoxin exposure. AKI often leads to long-term damage including acute kidney disease (AKD) and CKD. In adults, CKD is strongly associated with cardiovascular disease due to overlapping risk factors like hypertension and diabetes. Pediatric CKD also shows high hypertension prevalence, and early control via RAAS blockade and ambulatory BP monitoring is crucial (4–7).

Sepsis-associated acute kidney injury (S-AKI) is defined as a sudden decline in renal function occurring during sepsis or septic shock, characterized by an increase in serum creatinine or reduction in urine output as per KDIGO criteria. In patients with pre-existing CKD, this represents a superimposed acute insult that can accelerate kidney disease progression. Sepsis and septic shock are leading causes of mortality and are implicated in 25–75% of AKI cases globally. Septic AKI, once seen as transient, is now linked to progression toward CKD and end-stage kidney disease. In septic shock, blood pressure regulation is critical; current guidelines recommend targeting a MAP ≥65 mmHg, or 80–85 mmHg in chronic hypertensives, though vasopressors pose additional risks (8–13).

This study aims to identify risk factors and correlations between CKD and septic shock in both pediatric and adult populations.

## Methods

### Study design

We conducted a systematic scoping review in accordance with the Preferred Reporting Items for Systematic Reviews and Meta-Analyses (PRISMA) 2020 guidelines. The protocol for this review was prospectively registered on PROSPERO (Registration ID: CRD420251146866). The objective was to synthesize evidence on risk factors for CKD, hypertension, and their associations with S-AKI and septic shock in both adult and pediatric populations.

### Overview of the systematic literature review process

The methodology employed in this research is the Systematic Literature Review (SLR), aimed at identifying, assessing, and interpreting all relevant research findings related to the Risk Factors for CKD and Septic Shock with Hypertension in Adults and Children. The SLR process follows the PRISMA guidelines, which consists of the following stages:

- Identification: In this stage, a literature search is conducted to gather articles, journals, and other documents relevant to the research topic. The search is performed through electronic databases such as Google Scholar, PubMed, and Cochrane using predetermined keywords.
- Screening: After the identification stage, the search results are screened to remove duplicates and irrelevant articles. Articles that do not meet the inclusion criteria or are outside the scope of the research are eliminated at this stage.
- Eligibility: Articles that pass the screening stage are then evaluated for eligibility based on the established inclusion and exclusion criteria. Articles that do not provide sufficient data or are not relevant to the research focus are also eliminated at this stage.
- Inclusion: Articles that meet all criteria are included for further analysis. This stage results in a final list of literature that will be analyzed in depth in the research.

### Eligibility criteria (PECO framework)

- Population (P): Adults and children with pre-existing CKD and/or hypertension who developed sepsis or septic shock.
- Exposure (E): Presence of CKD, hypertension, albuminuria, and hemodynamic variables (mean arterial pressure [MAP], mean perfusion pressure [MPP]).
- Comparator (C): Patients without CKD or hypertension (where reported).
- Outcome (O):
- Primary Outcome: Incidence of S-AKI, defined as AKI occurring in the context of sepsis or septic shock based on KDIGO criteria.
- Secondary Outcomes: Progression to AKD, CKD progression, need for renal replacement therapy (RRT), in-hospital and 90-day mortality, and hemodynamic outcomes.

### Search strategy

A comprehensive search was conducted in PubMed/MEDLINE, Cochrane Library, and Google Scholar. The final search was performed on March 15, 2024. Equivalent search terms were adapted for Cochrane and Google Scholar. We included studies published in English or Indonesian, and additionally screened studies in other languages if an English abstract was available to minimize language bias.

### Study selection

Two reviewers independently screened all titles and abstracts using Rayyan AI. Full-text screening was conducted for potentially eligible articles. Discrepancies were resolved by consensus or arbitration by a third senior reviewer. The screening process and study selection are summarized in the updated PRISMA 2020 flow diagram, which includes the number of records at each stage (identification, screening, eligibility, and inclusion).

### Data extraction

After the literature selection process is completed, the next stage is data extraction from the selected articles. This process includes identifying and recording key information from each article relevant to the research objectives.

#### Search string

The literature search is conducted using various keywords relevant to the research topic. The keywords used are tailored to the databases accessed and include terms such as “chronic kidney disease”, “Septic Shock” and “Hypertension”.

#### Inclusion and exclusion criteria

##### Inclusion criteria

(1) articles published in reputable scientific journals; (2) publications from the last 20 years to ensure data relevance; and (3) articles available in either English or Indonesian.

##### Exclusion criteria

1) Articles that do not provide empirical data or concrete research findings.

2) Articles that are not fully accessible (only available as abstracts).

## Results

Our initial search yielded 762 records across PubMed, Cochrane Library, and Google Scholar. After removing 104 duplicates, 658 unique records were screened by title and abstract. Of these, 617 were excluded because they did not meet inclusion criteria (unrelated topic, wrong population, or non-empirical articles). A total of 41 full-text articles were assessed for eligibility, and 32 were excluded for the following reasons: pediatric case reports (n=6), narrative reviews or editorials (n=9), incomplete or inaccessible full text (n=7), and wrong outcomes (n=10).

**Figure 1:**
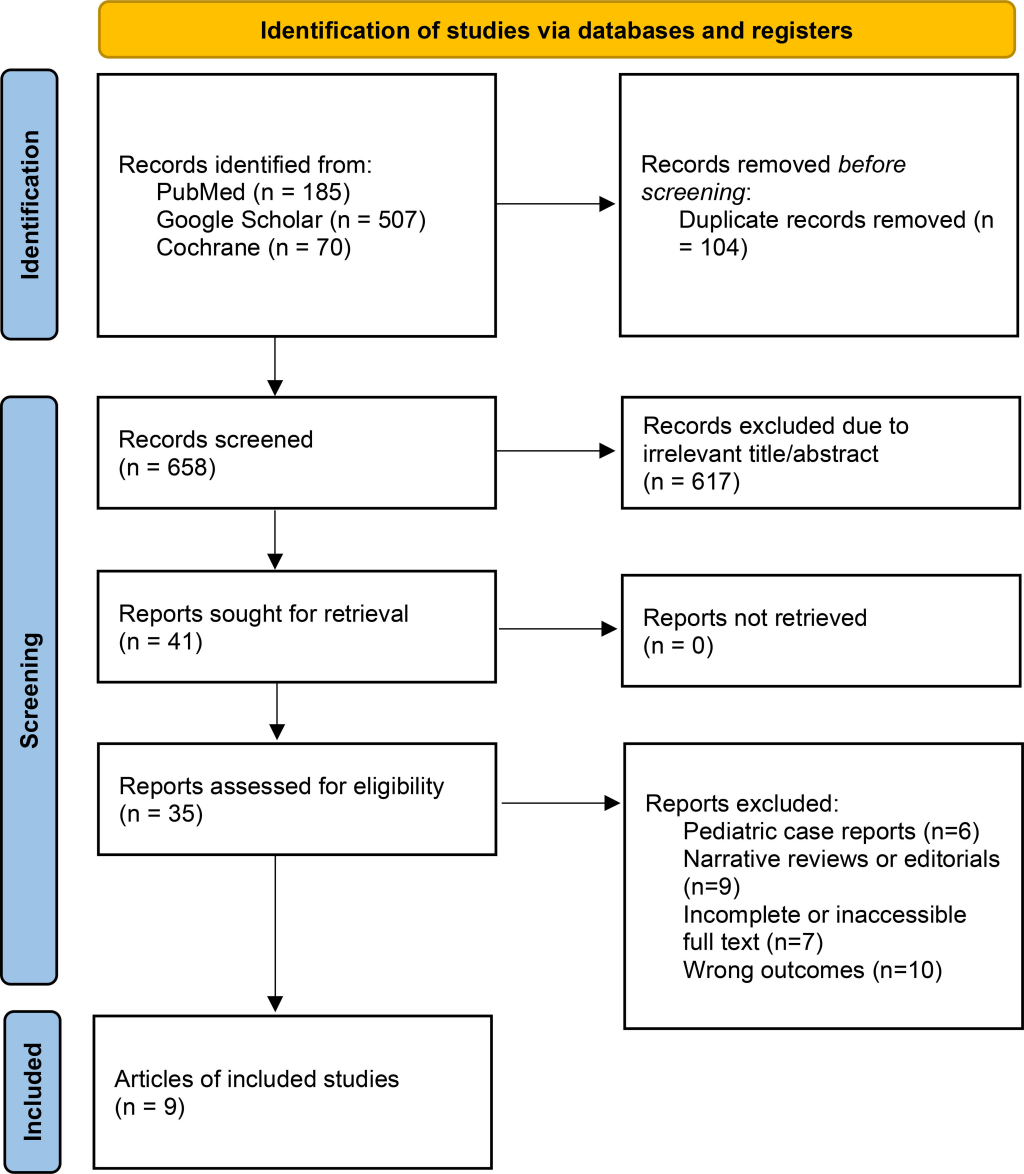
Systematic review diagram based on PRISMA (1).

Ultimately, 9 studies met all inclusion criteria (**Table 1**) their summarized data and effect estimates are presented in **Table 2**, and were included in the final qualitative synthesis (**Figure 1**).

**Table 1:** Included studies.

No	Author(s)	Year	Title of paper	Type of publication	Summary of finding
1.	Liu, J. et al. (14)	2020	Rates, predictors, and mortality of sepsis-associated acute kidney injury: a systematic review and meta-analysis	A systematic review and meta-analysis	A comprehensive systematic review and meta-analysis by Liu et al. (2020), titled “Rates, Predictors, and Mortality of Sepsis-Associated Acute Kidney Injury (S-AKI),” provides critical insight into the intersection between sepsis, AKI, and preexisting comorbidities such as hypertension and CKD. Drawing data from 47 studies encompassing 55,911 patients, the analysis revealed that approximately 48.73% of patients with sepsis developed AKI, with septic shock being the most significant clinical contributor. Importantly, the presence of preexisting hypertension and CKD were consistently identified as strong, independent predictors of S-AKI. Patients with these comorbidities were not only more likely to experience renal injury but also exhibited significantly higher mortality rates, particularly when dialysis was required. These findings are highly relevant to the present review, as they reinforce the bidirectional and compounding relationships between hypertension, CKD, and sepsis. Specifically, hypertension appears to exacerbate renal vulnerability in septic states, while underlying CKD impairs renal compensatory mechanisms, facilitating the progression to septic shock. Thus, the presence of hypertension and CKD should be viewed as both predisposing and prognostic factors in the development and clinical course of sepsis-associated complications.
2.	Huang M et al. (15)	2015	Association of kidney function and albuminuria with prevalent and incident hypertension: the Atherosclerosis Risk in Communities (ARIC) study	Cohort Study	The Atherosclerosis Risk in Communities (ARIC) study provides crucial evidence supporting the identification of high-risk patients for CKD progression and septic shock complications in hypertensive populations. The study’s key finding demonstrated that while all kidney markers were associated with prevalent hypertension, only albuminuria consistently predicted incident hypertension, establishing kidney damage as a more significant predictor than overall functional decline. This finding directly relates to risk factors for CKD and septic shock with hypertension by highlighting how albuminuria creates a pathophysiological cycle where initial kidney damage leads to hypertension development, which subsequently accelerates CKD progression. Furthermore, patients with concurrent hypertension and albuminuria represent a particularly vulnerable population for septic shock development, as their compromised renal microvascular integrity and reduced functional reserve impair hemodynamic responses during infectious stress. In pediatric populations, the presence of both hypertension and albuminuria indicates established kidney damage during critical developmental periods, predisposing children to more severe complications during septic episodes due to limited renal reserve capacity. The ARIC study findings emphasize albuminuria screening as an essential clinical tool for early identification of patients at elevated risk for both CKD progression and septic shock complications across all age groups, supporting targeted interventions to prevent adverse outcomes in hypertensive adults and children.
3.	Yamagata K et al. (16)	2007	Risk factors for chronic kidney disease in a community-based population: a 10-year follow-up study	Cohort Study	The 10-year community-based follow-up study involving 123,764 adults identified key risk factors for CKD development in the general population, providing crucial epidemiological evidence that directly supports CKD and septic shock risk stratification in hypertensive patients. This large-scale longitudinal investigation demonstrated how traditional cardiovascular risk factors, including hypertension, diabetes, and proteinuria, significantly predict CKD development over time. The study’s findings are particularly relevant to understanding septic shock vulnerability, as patients with established CKD risk factors show compromised renal hemodynamic responses and reduced functional reserve capacity. In clinical practice, these risk factors serve as early warning indicators for both CKD progression and increased susceptibility to septic complications in adults and children with hypertension, where the underlying kidney dysfunction creates a pathophysiological environment prone to acute decompensation during infectious stress.
4.	Bahrey, D. et al. (17)	2019	Prevalence and associated factors of chronic kidney disease among adult hypertensive patients in Tigray teaching hospitals: a cross-sectional study	Cross-sectional	A cross-sectional study of 578 adult hypertensive patients in Tigray teaching hospitals revealed a considerably high prevalence of CKD (22.1%), establishing critical risk factors that directly support CKD and septic shock vulnerability assessment in hypertensive populations. The study identified age >60 years [AOR 1.43], uncontrolled hypertension [AOR 9.45], overweight/obesity [AOR 7.42], dyslipidemia [AOR 13.75], and diabetes mellitus [AOR 2.14] as significant independent predictors of CKD development. These findings are particularly relevant to septic shock risk stratification, as patients with multiple CKD risk factors demonstrate compromised renal reserve and increased susceptibility to AKI during infectious episodes. The study’s identification of uncontrolled hypertension as a major risk factor emphasizes the importance of blood pressure management in preventing both CKD progression and subsequent septic complications in adults and children with hypertensive disease.
5.	Peerapornratana S et al. (18)	2020	Sepsis-Associated Acute Kidney Disease	Observational cohort	The study on Sepsis-Associated Acute Kidney Disease (AKD), published in BMC Nephrology (2020), offers valuable clinical insights into the intermediate trajectory between AKI and CKD in patients with sepsis. Conducted among 1,341 patients hospitalized for septic shock, the study found that 26.9% of those who developed AKI went on to experience AKD—defined as sustained renal dysfunction lasting more than 7 days following the initial AKI event. Multivariable analysis identified several independent predictors of AKD, including older age, underlying CKD, high baseline serum creatinine, and persistent hemodynamic instability. Notably, underlying CKD was one of the strongest risk factors, with affected individuals significantly more likely to experience prolonged renal dysfunction and adverse outcomes. In-hospital mortality was markedly higher in the AKD group compared to patients whose renal function recovered rapidly (31.4% vs 17.8%). This finding is highly relevant to the current systematic review, as it highlights the synergistic burden of preexisting CKD and sepsis, especially in the presence of hypertension, which often coexists and contributes to renal vulnerability. The progression from AKI to AKD in septic patients reflects an important pathophysiological continuum where immune dysregulation, impaired renal autoregulation, and chronic vascular injury converge—underscoring the need for early recognition and risk stratification in hypertensive and CKD patients at risk of septic shock.
6.	Li, L. et al. (19)	2024	Association between the mean perfusion pressure and the risk of acute kidney injury in critically ill patients with sepsis: a retrospective cohort study	Retrospective Cohort Study	A retrospective cohort study investigating the association between mean perfusion pressure (MPP) and AKI in critically ill septic patients found that lower MPP levels are significantly associated with an increased incidence of AKI. Specifically, the study revealed that a lower MPP (e.g., below 63 mmHg, or below 60 mmHg in some analyses) was a significant risk factor for AKI development within 7 days of ICU admission. Each 1 mmHg increase in MPP was associated with a decrease in AKI incidence, and patients maintained at higher MPP levels exhibited a lower risk of AKI and improved in-hospital survival rates. This suggests that optimizing MPP is crucial for preventing AKI in this vulnerable patient population.
7.	Neyra JA et al. (20)	2019	Impact of Acute Kidney Injury and CKD on Adverse Outcomes in Critically Ill Septic Patients	Cohort Study	A retrospective cohort study by Heung et al. (2019) investigated the joint impact of AKI and pre-existing CKD on outcomes in 2,632 critically ill adults admitted with severe sepsis or septic shock. The study stratified patients based on CKD status (eGFR ≥60 vs. 15–59 ml/min/1.73 m²) and AKI severity according to KDIGO criteria. Results revealed that AKI occurred in 57% of patients and CKD was present in 46% at baseline. Critically, patients with AKI stage ≥2 regardless of CKD status had significantly higher 90-day mortality. Specifically, adjusted hazard ratios for mortality were 2.4 (95% CI: 1.9–3.1) in patients without CKD and 2.2 (95% CI: 1.7–2.9) in those with CKD, compared to patients without AKI. Interestingly, CKD alone or CKD combined with stage 1 AKI did not significantly increase mortality. These findings highlight a synergistic burden of AKI and advanced CKD during sepsis, where the presence of moderate-to-severe renal injury, rather than CKD alone, was the critical determinant of adverse outcomes. In the context of the current review, this study reinforces that hypertension-related CKD, a known comorbidity in septic patients, amplifies vulnerability to AKI and is associated with poorer outcomes when coupled with sepsis-induced renal insults. The data underscore the need for rigorous monitoring and prevention of AKI progression in hypertensive patients with underlying CKD who are at risk for or already experiencing septic shock.
8.	Patel M et al. (21)	2024	A reappraisal of risk factors for hypertension after pediatric acute kidney injury.	Observational Study	This recent pediatric study highlights that survivors of AKI are at a significantly elevated risk of developing new-onset hypertension. This finding is directly relevant to the current review as it underscores a bidirectional pathophysiological relationship—where AKI may lead to hypertension, which in turn accelerates the progression to CKD. Collectively, these interconnected conditions heighten susceptibility to septic decompensation, particularly among pediatric patients with reduced nephron endowment.
9.	Kim JS et al. (22)	2018	One–Year Progression and Risk Factors for the Development of Chronic Kidney Disease in Septic Shock Patients with Acute Kidney Injury: A Single-Centre Retrospective Cohort Study	Retrospective Cohort Study	In this retrospective study involving adult patients with septic shock, only 27% achieved full renal recovery after one year, while 6% progressed to CKD. Among those with stage 1 AKI, 10% developed CKD and the one-year mortality rate was 13%. In contrast, patients with stage 2 and 3 AKI had lower CKD progression rates (6%) but markedly higher mortality rates of 42% and 47%, respectively. Identified risk factors for CKD progression included advanced age, female sex, diabetes mellitus, low hemoglobin levels, and elevated serum creatinine at discharge. These findings emphasize the complex interplay between AKI severity, renal recovery, and long-term outcomes in septic patients, and underscore the need for close monitoring of high-risk individuals.

**Table 2:** Summary of included studies and effect estimates.

Author/Year	Design	N	Exposure/risk factor	Primary/key outcomes	Effect size/key findings
Liu et al. (2020) (14)	Systematic review & meta-analysis	55,911	Septic shock, HTN, DM	S-AKI incidence	Septic shock OR 2.88 (2.36–3.52); HTN OR 1.43 (1.20–1.70); DM OR 1.59 (1.47–1.71)
Huang et al. (2015) (15)	Prospective cohort (ARIC)	4,378 baseline	Albuminuria (ACR quintiles)	Incident HTN (9.8 y follow-up)	HR 1.28 (1.10–1.49) for highest vs lowest quintile; even mild ACR elevation associated with incident HTN
Yamagata et al. (2007) (16)	10-year community cohort	123,764	HTN, proteinuria, metabolic risk factors	Incident CKD	>2× HR for uncontrolled HTN or proteinuria; CKD incidence 19.2%
Bahrey et al. (2019) (17)	Cross-sectional	578	Uncontrolled BP, dyslipidemia, obesity	CKD prevalence	AOR 9.45 (1.34–14.73) uncontrolled HTN; AOR 13.7 (5.69–33.2) dyslipidemia
Peerapornratana et al. (2020) (18)	Prospective observational (secondary analysis of RCT cohort)	598	AKI stage, baseline CKD, demographics	AKD development, relapse, 7-day mortality	26.9% developed AKD; only 9.3% recovered renal function before discharge; relapse occurred in 14.2% with early reversal; baseline CKD and male sex ↑ risk
Li et al. (2024) (19)	Retrospective ICU cohort	5,867	Mean perfusion pressure (MPP <63 mmHg)	AKI within 7 days, survival	OR 0.71 (0.61–0.83) for MPP ≥ 63 mmHg; each +1 mmHg MPP ↓ AKI risk 2%; higher survival in high-MPP group
Neyra et al. (2019) (20)	Cohort	2,632	CKD ± AKI	90-day mortality, CKD progression	HR 2.4 (1.9–3.1) for AKI ≥ 2 without CKD; HR 2.2 (1.7–2.9) for AKI ≥ 2 with CKD
Patel et al. (2024) (21)	Pediatric cohort	410	AKD, KRT, CHD	New-onset HTN >90 days	19% developed HTN; multivariable model: AKD, KRT, CHD significant predictors
Kim et al. (2018) (22)	Single-centre retrospective cohort	2,208	AKI stage severity	CKD progression, 1-year mortality	CKD progression 6%; mortality 42–47% for stage 2–3 AKI vs 13% stage 1

### Bias assessment

A risk of bias assessment was conducted for all nine included studies using appropriate tools according to study design: JBI Critical Appraisal Tools for observational studies and AMSTAR 2 for the systematic review and meta-analysis. Most of the studies were rated as low to moderate risk of bias. Common strengths across studies included clearly defined inclusion criteria, appropriate statistical analyses, and clinically relevant outcomes. However, several cohort and cross-sectional studies demonstrated potential limitations such as lack of adjustment for confounders, unclear follow-up duration, or insufficient detail on blinding and selection procedures (**Table 3**).

**Table 3:** Risk of bias assessment.

No.	Author (Year)	Study type	Risk of bias	Notes
1.	Liu et al. (2020) (14)	Systematic Review & Meta-Analysis	Low (AMSTAR-2)	Comprehensive search and analysis; lacks protocol registration
2.	Huang et al. (2015) (15)	Cohort Study	Low (JBI)	Strong design, large sample size, good confounder control
3.	Yamagata et al. (2007) (16)	Cohort Study	Moderate (JBI)	Longitudinal but limited detail on loss to follow-up
4.	Bahrey et al. (2019) (17)	Cross-Sectional	Moderate (JBI)	Prone to selection and recall bias; no temporal relationship
5.	Peerapornratana et al. (2020) (18)	Observational Cohort	Low (JBI)	Good methodology; clear endpoints and statistical adjustment
6.	Li et al. (2024) (19)	Retrospective Cohort	Moderate (JBI)	Strong analysis, but retrospective design limits causality
7.	Neyra et al. (2019) (20)	Cohort Study	Low (JBI)	Robust statistical control and clear outcome definitions
8.	Patel et al. (2024) (21)	Observational (Pediatric)	Moderate (JBI)	Appropriate design but limited follow-up on long-term BP/CKD
9.	Kim et al. (2018) (22)	Retrospective Cohort	Moderate (JBI)	Good data analysis but lacks control for some confounders

### Study characteristics

Nine studies published between 2007 and 2024 were included, encompassing more than 185,000 participants across various settings, including community-based cohorts, retrospective ICU analyses, and one prospective observational study nested within a randomized trial (14–22). Six studies focused exclusively on adult populations, one included both adults and children, one was a pediatric cohort, and one was a systematic review and meta-analysis synthesizing 47 observational studies (14).

### Primary outcome: incidence of sepsis-associated AKI

The incidence of S-AKI among septic patients ranged from 30% to 57%, with the highest rates observed in septic shock (14, 20, 22). In the meta-analysis including 55,911 patients, septic shock was the most potent risk factor for S-AKI (OR 2.88, 95% CI 2.36–3.52) (14). Prevalent CKD was reported in 46% of critically ill patients, and the combination of CKD and AKI significantly increased 90-day mortality (HR 2.2, 95% CI 1.7–2.9) (20).

### Hemodynamic targets (MAP and MPP)

One large retrospective cohort of 5,867 septic ICU patients demonstrated that lower MPP (<63 mmHg) was associated with a significantly higher incidence of AKI (87.6% vs. 78.3%, p<0.001) and higher in-hospital mortality (19). Each 1 mmHg increase in MPP conferred a 2% reduction in AKI risk (OR 0.98, 95% CI 0.97–0.99), supporting MPP as a potentially modifiable target in sepsis management (19).

### CKD and hypertension as risk factors

Several consistent risk factors for CKD emerged across studies, including age, hypertension, diabetes mellitus, proteinuria/albuminuria, obesity, and dyslipidemia (2–4). Yamagata et al. reported that treated hypertension and systolic BP >160 mmHg were associated with more than a twofold increase in CKD risk in females (3). Similarly, albuminuria was shown to be a strong predictor of both prevalent and incident hypertension, even at mildly elevated levels (2). Among hypertensive patients, uncontrolled blood pressure, overweight/obesity, and dyslipidemia were strongly associated with CKD (AOR 7.42–13.74) (4).

### Progression to AKD and CKD

The continuum from AKI to AKD and CKD was highlighted in several studies (18, 20, 22). Among patients with septic shock and AKI, only 27% regained baseline renal function within one year, while 6–10% progressed to CKD despite mild initial AKI stages (22). In the study of AKD, 26.9% of patients developed persistent renal dysfunction, and fewer than 10% of those recovered before discharge (18). These findings underscore the need for vigilant follow-up after discharge, particularly in patients with pre-existing CKD, diabetes, or high creatinine levels at discharge (22).

### Mortality and long-term outcomes

Mortality among patients with sepsis-associated AKI (S-AKI) remained high across the included studies, ranging from 13% in stage 1 AKI to >40% in stage 2–3 AKI at one year (22). The presence of both CKD and AKI amplified the risk of death, with adjusted hazard ratios for 90-day mortality reaching 2.2 (95% CI 1.7–2.9) in patients with CKD and stage ≥2 AKI compared to those without kidney disease (20). Patients with persistent AKD after sepsis had particularly poor outcomes, with in-hospital mortality of 19.9% and low rates of renal recovery (9.3%) before discharge (18).

Long-term outcomes showed that renal function recovery was incomplete in a substantial proportion of patients. Only about 27% of patients with septic shock–associated AKI achieved full renal recovery by 12 months, while 6–10% developed CKD regardless of initial AKI severity (22). These findings suggest that the risk of progression to CKD persists even after apparent clinical improvement.

Pediatric survivors of AKI also face long-term consequences, including new-onset hypertension (19%) and increased risk for CKD progression (21). These data reinforce the need for structured post-discharge follow-up programs, early nephrology referral, and surveillance of kidney function and blood pressure in both adult and pediatric populations recovering from septic AKI.

### Pediatric findings

Pediatric data remain limited but suggest that children surviving AKI are at substantial risk of developing new-onset hypertension within 90 days (19%) (21). Risk factors included AKD, need for kidney replacement therapy, congenital heart disease, and prior solid organ transplantation (21). These findings highlight the importance of early blood pressure surveillance and nephrology follow-up in pediatric AKI survivors.

## Discussion

This systematic review provides comprehensive evidence that CKD, hypertension, and sepsis form an interconnected pathophysiological triad that substantially impacts morbidity and mortality in critically ill patients. Across the nine included studies, pre-existing CKD and hypertension consistently emerged as independent predictors for S-AKI, adverse hemodynamic profiles, and worse survival outcomes (14–16, 20, 22, 23).

Several large-scale cohort studies and meta-analyses confirmed that patients with CKD are highly susceptible to developing S-AKI, and that failure to fully recover renal function after the acute episode may lead to AKD or progression to more advanced stages of CKD (14, 18, 22, 24). Importantly, Liu et al. demonstrated that nearly half of patients with sepsis developed AKI, and both CKD and hypertension were among the strongest predictors of adverse outcomes (14). This underscores the synergistic effect of CKD and hypertension in reducing renal reserve and amplifying vulnerability to septic insults (25).

Key risk factors for CKD development and progression—such as uncontrolled blood pressure, albuminuria, diabetes, dyslipidemia, and obesity—were consistently reported across studies (15–17). Hypertension was identified as the most potent modifiable risk factor, directly driving glomerular injury through intraglomerular hypertension and hyperfiltration. Even mildly elevated albuminuria was associated with future hypertension and CKD, emphasizing its utility as a screening biomarker for early intervention (15, 26). Metabolic comorbidities and advanced age further contribute to microvascular dysfunction, oxidative stress, and reduced nephron reserve, which together increase susceptibility to S-AKI and poor recovery following sepsis (16, 17, 22, 27).

Hemodynamic optimization plays a central role in mitigating S-AKI risk. The retrospective cohort by Li et al. reported that patients with MPP ≥63 mmHg had significantly lower AKI incidence and improved survival (19). These findings support individualized perfusion targets, particularly for patients with chronic hypertension, in whom higher MAP goals may be beneficial. Nevertheless, causality cannot be inferred, and randomized trials are needed to determine optimal hemodynamic thresholds (29).

Mortality remained unacceptably high among patients with S-AKI, particularly in those with advanced AKI stages or concurrent CKD (20, 22). Long-term follow-up data revealed that fewer than one-third of patients achieved full renal recovery by 12 months, and 6–10% progressed to CKD despite apparent initial improvement (22, 30). Pediatric survivors also demonstrated a 19% incidence of new-onset hypertension following AKI, highlighting the need for post-discharge surveillance and early nephrology referral in this population (21, 30).

Several clinical implications emerge from these findings. First, early identification of high-risk patients using baseline kidney function and albuminuria screening is critical for risk stratification (15, 26). Second, individualized hemodynamic targets, including consideration of higher MAP or MPP goals for hypertensive patients, may help preserve renal function (19, 28). Third, structured follow-up programs should be implemented to monitor renal recovery, blood pressure control, and CKD progression in both adults and children following sepsis (21, 30).

This review has several limitations. Most included studies were observational, introducing potential confounding and precluding causal inference. Definitions of AKI, AKD, and sepsis varied across studies, and significant heterogeneity was present in study designs, settings, and outcome reporting (23). The small number of pediatric-focused studies limits generalizability to younger populations, and no pooled meta-analysis could be performed due to heterogeneity. Despite these limitations, the consistency of associations across diverse populations strengthens the reliability of our conclusions (24, 30).

Future research should focus on well-designed prospective studies to define optimal blood pressure and perfusion targets, identify early biomarkers for S-AKI risk stratification, and evaluate interventions to improve renal recovery after sepsis (23, 29). Pediatric-specific trials are urgently needed to establish evidence-based guidelines for this vulnerable group (21, 31).

In conclusion, CKD and hypertension markedly increase the risk and severity of sepsis-related renal complications. Early recognition of at-risk patients, individualized hemodynamic management, and vigilant long-term follow-up represent key strategies to improve outcomes. A multidisciplinary approach involving nephrology, critical care, and pediatrics is essential to mitigate preventable morbidity and mortality in this high-risk population.

### Strengths and limitations

This review adhered to PRISMA 2020 guidelines, specified a PECO framework, and extracted effect sizes from all included studies. However, most of the included studies were observational, which introduces residual confounding. Significant heterogeneity exists in defining AKI, AKD, and fluid management protocols, precluding formal meta-analysis. Although we expanded the search to multiple databases, language bias remains possible as we primarily included English and Indonesian full texts.

### Future directions

Future research should focus on prospective, multicenter studies to establish optimal hemodynamic targets (MAP and MPP) and fluid strategies in patients with CKD and hypertension. Additionally, standardized definitions for AKD and CKD progression are needed to enable pooling of data across studies. Dedicated pediatric cohorts are urgently required to guide best practices for this vulnerable population.

## Conclusion

This systematic review highlights the complex interplay between CKD, hypertension, and septic shock in both adults and pediatric populations. The evidence consistently demonstrates that pre-existing hypertension and CKD are not only significant risk factors for (S-AKI, but also contribute to poor renal recovery and increased mortality during septic episodes. Key predictors such as albuminuria, advanced age, diabetes mellitus, and uncontrolled blood pressure were repeatedly identified across studies as drivers of both CKD progression and sepsis vulnerability. Additionally, impaired hemodynamic regulation—particularly reduced MPP—was shown to exacerbate renal injury in septic patients. Careful fluid management, individualized blood pressure targets, and early identification of high-risk patients using clinical markers like albuminuria are essential to improving outcomes. In pediatric patients, early-onset AKI and hypertension require long-term surveillance due to their potential to trigger lifelong renal compromise. Overall, integrated, multidisciplinary strategies are critical for optimizing care in this high-risk population, minimizing the progression to end-stage kidney disease, and reducing sepsis-related mortality.

## Data Availability

The original contributions presented in the study are included in the article/supplementary material. Further inquiries can be directed to the corresponding author.
